# Comment on Dutheil, Mondillon, and Navel (2020): the importance of adjustment disorders and resilience

**DOI:** 10.1017/S003329172000344X

**Published:** 2020-09-08

**Authors:** Roel Van Overmeire

**Affiliations:** Mental Health and Wellbeing Research Group, Vrije Universiteit Brussel, Laarbeeklaan 103, 1090 Jette, Belgium

In a commentary, Dutheil, Mondillon, and Navel (2020) state there is a high risk that post-traumatic stress disorders (PTSDs) will increase after the current pandemic. They call it ‘a second tsunami’ after the current one of COVID-19. Though there is undoubtedly a risk of more mental health problems in the population, there are a few remarks to be made on there being a ‘second tsunami of PTSD’.

First, it is questionable if PTSD is even the proper diagnoses for most cases during the COVID-19 pandemic. A recent perspective in the *New England Journal of Medicine* even stated that the diagnosis of PTSD was not supported in the DSM-5 (Pfefferbaum & North, 2020). After all, the DSM-5's inclusion criteria for PTSD are not compatible with the situations that often occur during the COVID-19 pandemic. While the authors state that PTSD is causes by a ‘terrifying event outside the normal range of usual human experience’, the diagnostic features in criterion A of PTSD actually refer to life-threatening events, or events where someone is seriously wounded (American Psychiatric Association, 2014). Furthermore, from a biological standpoint, it is unclear for many situations how a ‘fight-or-flight’ mechanism would be triggered, although such a mechanism is an essential part of a possible development of PTSD. For example, when being threatened with a gun by a robber, someone might fight, someone might try to flee, or if this does not happen, someone might freeze (Van der Kolk, 2015). It is unclear how this mechanism would work in the case of healthcare workers who must treat people who are dying of a disease, or someone in his/her 20s being infected – as the overwhelming majority of adolescents survive COVID-19. If this is indeed a life-threatening situation, almost every situation with a disease is life-threatening (e.g. the flu).

Because of such questions, some authors have stated that an adjustment disorder (AD) might be a more suitable diagnosis (Kazlauskas & Quero, 2020). ADs are similar to PTSD, only they are void of the biological reactions of PTSD (e.g. the fight-or-flight reaction) and do not require a stressor that is life-threatening or traumatic (Maercker, Einsle, & Köllner, 2007). AD can be diagnosed after stressors such as losing one's job (applicable during an economic recession such as now), very stressful work (e.g. healthcare workers in a hospital) or even serious illness (such as COVID-19 for risk populations) (Maercker et al., 2007). Therefore, AD is very fitting for today's context. Keeping this in mind, it is not surprising that Kazlauskas and Quero (2020) have warned for a worldwide spread of ADs due to the COVID-19 pandemic. Yet, research remains sparse on AD, despite it being very common in clinical practice (O'Donnell et al., 2016). However, based on the information available, it seems more likely there would be a second tsunami of AD.

A second remark to be made is that people are far more resilient than is often considered in comments on mental health consequences of COVID-19. While the COVID-19 pandemic is unprecedented, the comparison between the mental health consequences of terrorism and COVID-19 has been made (DePierro, Lowe, & Katz, 2020). While indeed there are similarities in the possible mental health problems caused by both events, there are also lessons to be learned on resilience. Durodié and Wainwright (2019) found when studying terrorism studies using PTSD-scales, that first, many of such studies systematically overestimate PTSD, but second, that people are far more resilient than we would expect. In fact, while during the first month there might be acute emotional reactions, these seldom develop into PTSD (Durodié & Wainwright, 2019).

Bonanno (2008) made a similar observation more than a decade ago: people are far more resilient than would be expected based on the conclusions often drawn by researchers. This does not mean that traumatic events are rare – in fact, most people experience a traumatic event at one point in their life – but only a small proportion develop PTSD, and even fewer chronic PTSD (meaning, more than 1 year) (Friedman, Keane, & Resick, 2016).

To conclude, while there is a chance of an increase of mental health problems, it is first unlikely that PTSD would be the proper diagnosis, and second, if it is, it will probably not be a tsunami of PTSD that comes toward us. It would be a small wave, at best. This does not mean that we should ignore the possible problems that might arise. We must remain observant, and perform studies, using proper methodology, but panic because of possible huge increases in PTSD do not seem warranted.
